# Supplementary material for: Opuntia dillenii as a Nutraceutical and Dietary Resource for Disease Prevention and Management: A Systematic Review
Source: Nutrients. 2025 Dec 14;17(24):3915. doi: 10.3390/nu17243915 (PMC12735451; doi:10.3390/nu17243915)
Supplement: Supplementary file 1 [file nutrients-17-03915-s001.zip › nutrients-4005048-supplementary.pdf]

Table S1. Excluded records

Author	Title	Publication year	DOI	Exclusion criteria
Ayyar	Control of prickly-pear by the cochineal insect	1931	10.1038/128117b0	Ethnobotanics
Vanoverbeek et al.	Use of 2,4-Dichlorophenoxyacetic Acid As a Selective Herbicide in the Tropics	1946	10.1126/science.103.2677.472	Ethnobotanics
Nair et al.	Isolation of Isoquercitrin from Flowers of *Opuntia dillenii*	1964	-	Chemical characterization
Oakes	Herbicidal Control of *Opuntia*	1971	-	Ethnobotanics
Sciuto et al.	Pigments of Centrospermae .15. Betanidin Glucosylation in *Opuntia*-Dillenii	1972	10.1016/S0031-9422(00)88386-7	Chemical characterization
Srivastava et al.	Arabinogalactan from Pods of *Opuntia*-Dillenii	1974	10.1055/s-0028-1097919	Chemical characterization
Meyer et al.	Cactus Alkaloids .43. Beta-Phenethylamines from the Cactus Genus *Opuntia*	1980	10.1016/0031-9422(80)87057-9	Chemical characterization
Doust et al.	Fertility Relationships in Closely Related Taxa of Oxalis, Section Corniculatae	1981	10.1139/b81-312	Ethnobotanics
Nobel	Influences of Photosynthetically Active Radiation on Cladode Orientation, Stem Tilting, and Height Of Cacti	1981	10.2307/1936997	Ethnobotanics
Doust el al.	Biology of Canadian Weeds .71. Oxalis-Stricta L, Oxalis-Corniculata L, Oxalis-Dillenii Jacq Ssp Dillenii and Oxalis-Dillenii Jacq Ssp Filipes (Small) Eiten	1985	10.4141/cjps85-090	Ethnobotanics
Marshall	A Review of the Biology and Control of Selected Weed Species in the Genus Oxalis - Oxalis-Stricta L, Oxalis-Latifolia Hbk and Oxalis-Pes-Caprae L	1987	10.1016/0261-2194(87)90068-8	Review

Ellenberg	*Opuntia*-Dillenii as a Problematic Neophyte in The Yemen-Arab-Republic	1989	10.1016/S0367-2530(17)30390-0	Ethnobotanics
Watson	Variation and systematics of European members of Oxalis L. section corniculatae DC	1990	-	Ethnobotanics
Moran et al.	Biological-Control of Cactus Weeds of Minor Importance in South-Africa	1991	10.1016/0167-8809(91)90138-N	Ethnobotanics
Valido et al.	Frugivory and Seed Dispersal by the Lizard Gallotia-Galloti (Lacertidae) in a Xeric Habitat of the Canary-Islands	1994	10.2307/3545778	Ethnobotanics
Deshpande et al.	Cactus (*Opuntia*-Dillenii Grahm) Stem - A New Source of Energy	1994	10.1016/0378-7753(94)80060-X	Non-food application
Cavers	The biology of Canadian weeds: contributions 62-83	1995	-	Ethnobotanics
Amador	Ecología de la Dispersión de Semillas Por los Lagartos Endémicos Canarios (G: Gallotia, Lacertidae)	1999	-	Ethnobotanics
Lima et al.	Record of host plants (Cactaceae) and new dissemination strategy of Diaspis echinocacti (Bouché) (Hemiptera: Diaspididae), prickly-pear-scale, in the States of Pernambuco and Alagoas, Brazil	2001	10.1590/S1519-566X2001000300025	Ethnobotanics
Shekhar et al.	Assessment of livestock grazing pressure in and around the elephant corridors in Mudumalai Wildlife Sanctuary, south India	2001	10.1023/A:1013285910650	Ethnobotanics
Liu et al.	Effects of Sheng-Mai injection on the PRPP synthetase activity in BFU-Es and CFU-Es from bone marrows of mice with benzene-induced aplastic anemia	2001	10.1016/S0024-3205(01)01224-3	No *Opuntia dillenii*
Qiu et al.	Constituents with radical scavenging effect from *Opuntia dillenii*:: Structures of new α-pyrones and flavonol glycoside	2002	10.1248/cpb.50.1507	Chemical characterization
Gupta et al.	Antispermatogenic effect and chemical investigation of *Opuntia dillenii*	2002	10.1076/phbi.40.6.411.8437	Non-food application

Qiu et al.	The isolation and identification of a new α-pyrone from *Opuntia dillenii*	2003	-	Chemical characterization
Murugan et al.	Purification and characterization of cinnamyl alcohol-NADPH-dehydrogenase from the leaf tissues of a basin mangrove Lumnitzera racemosa Willd.	2004	-	No *Opuntia dillenii*
Qiu et al.	Chemical constituents of *Opuntia dillenii*	2005	-	Chemical characterization
Jiang et al.	Two novel C29-5β-sterols from the stems of *Opuntia dillenii*	2006	10.1016/j.steroids.2006.09.005	Chemical characterization
Liang et al.	Microwave-assisted technology for extracting *Opuntia* polysaccharide	2006	-	Chemical characterization
Tao et al.	Isolation and purification of polysaccharides from *Opuntia dillenii* and their properties	2006	-	Chemical characterization
Grøstad et al.	Yellow-flowering Oxalis species in Norway	2006	-	Ethnobotanics
Diaz-Medina et al.	Chemical characterization of *Opuntia dillenii* and *Opuntia* ficus indica fruits	2007	10.1016/j.foodchem.2006.06.064	Chemical characterization
Liu et al.	Determination of trace elements in new food sources by flame atomic absorption spectrophotometry	2007	-	Chemical characterization
Qiu et al.	Two new α-pyrones and other components from the cladodes of *Opuntia dillenii*	2007	10.1007/BF02977624	Chemical characterization
Cervera et al.	Photosynthesis and optimal light microhabitats for a rare cactus, Mammillaria gaumeri, in two tropical ecosystems	2007	10.1111/j.1744-7429.2007.00311.x	Ethnobotanics
Negron-Ortiz	Chromosome numbers, nuclear DNA content, and polyploidy in Consolea (Cactaceae), an endemic cactus of the Caribbean islands	2007	10.3732/ajb.94.8.1360	Ethnobotanics
Padilla et al.	Prey size selection of insular lizards by two sympatric predatory bird species	2007	10.3161/068.042.0211	No *Opuntia dillenii*

Badami et al.	In vitro antioxidant activity of thirteen medicinal plants of India's Western Ghats	2007	10.1080/13880200701215141	Only antioxidant
Boehm et al.	"*Opuntia dillenii*" - An Interesting and Promising Cactaceae Taxon	2008	-	Ethnobotanics
Chang et al.	The protective effect of *Opuntia dillenii* Haw fruit against low-density lipoprotein peroxidation and its active compounds	2008	10.1016/j.foodchem.2007.06.017	Only antioxidant
Chang et al.	The protective effect of *Opuntia dillenii* Haw fruit against low-density lipoprotein peroxidation and its active compounds	2008	10.1016/j.foodchem.2007.06.017	Only antioxidant
Ayyanar et al.	Herbal medicines for wound healing among tribal people in Southern India: Ethnobotanical and scientific evidences	2009	-	Ethnobotanics
Firmino De Azevedo el al.	Effect of fruit maturation stage and substrate in the germination of *Opuntia dillenii* (Cactaceae) seeds	2009	10.17660/ActaHortic.2009.811.47	Ethnobotanics
Nesom	Again: Taxonomy of yellow-flowered caulescent oXALlS (Oxalidaceae) in Eastern North America	2009	-	Ethnobotanics
Liu et al.	Supercritical carbon dioxide extraction of seed oil from *Opuntia dillenii* Haw. and its antioxidant activity	2009	10.1016/j.foodchem.2008.09.049	Only antioxidant
Syu et al.	Survey of functional food molecules in prickly pear cactus, bitter gourd, and guava for the dietary management of the Type II diabetes	2009	10.1096/fasebj.23.1_supplement.900.4	Review
Abbasi et al.	6-Hydroxymethyl-4-methoxy-2H-pyran-2-one (Opuntiol)	2010	10.1107/S1600536809053860	Chemical characterization
Touil et al.	Physico-Chemical Characterisation of *Opuntia dillenii* Fruit	2010	10.2202/1556-3758.1601	Chemical characterization
Wei et al.	Study on the separation and purification of total flavone in cactus by macroporous adsorption resins	2010	-	Chemical characterization

Fernanda et al.	Environmental regulation of carbon isotope composition and crassulacean acid metabolism in three plant communities along a water availability gradient	2010	10.1007/s00442-010-1724-z	Ethnobotanics
Huang et al.	Determinants of the geographical extent of invasive plants in China: effects of biogeographical origin, life cycle and time since introduction	2010	10.1007/s10531-009-9751-y	Ethnobotanics
Rahmatullah et al.	A pharmacological and phytochemical evaluation of medicinal plants used by the Harbang clan of the Tripura tribal community of Mirsharai area, Chittagong district, Bangladesh	2010	10.1089/acm.2009.0497	Ethnobotanics
Sharma et al.	Survey report of medicinal plant used in folk medicine in tribal areas of Pandhurna, District Chhindwara (Madhya Pradesh)	2010	-	Ethnobotanics
Loganayaki et al.	In vitro Antioxidant Properties of Indigenous Underutilized Fruits	2010	10.1007/s10068-010-0102-2	Only antioxidant
Jeannin	Exotic medicinal plants (5): *Opuntia dillenii* (Ker-Gawl.) Haw; *Opuntia* ficus-indica (L.) Mill.	2010	10.1159/000320785	Review
Ayyanar et al.	Ethnobotanical survey of medicinal plants commonly used by Kani tribals in Tirunelveli hills of Western Ghats, India	2011	10.1016/j.jep.2011.01.029	Ethnobotanics
Foxcroft el al.	Expanding the conceptual frameworks of plant invasion ecology	2011	10.1016/j.ppees.2011.03.004	Ethnobotanics
Murugesan et al.	Ethnobotanical study of medicinal plants used by villagers in Kolli Hills of namakkal district of Tamil Nadu, India	2011	-	Ethnobotanics
Padron et al.	Integration of invasive *Opuntia* spp. by native and alien seed dispersers in the Mediterranean area and the Canary Islands	2011	10.1007/s10530-010-9872-y	Ethnobotanics
Rahmatullah et al.	A survey of medicinal plants used by garo and non-garo Traditional Medicinal Practitioners in Two Villages of Tangail District, Bangladesh	2011	-	Ethnobotanics

Ravikumar et al.	Ethnobotanical survey of coastal medicinal plants along the Palk Strait coast of south India	2011	10.1080/10496475.2011.584823	Ethnobotanics
Chen et al.	Wound healing effects of cactus extracts on second degree superficial burned mice	2011	-	Non-food application
Wang et al.	Chemical constituents from *Opuntia dillenii*	2012	-	Chemical characterization
Parthasarathy et al.	Invasive Alien Plants in Tropical Forests of the South-eastern Ghats, India: Ecology and Management	2012	10.1079/9781845939076.0162	Ethnobotanics
Verma et al.	Ethno-medicinal uses of some plants of Kanag hill in Shimla, Himachal Pradesh, India	2012	-	Ethnobotanics
Bajaj et al.	Fertility suppression in male albino rats by administration of methanolic extract of *Opuntia dillenii*	2012	10.1111/j.1439-0272.2011.01220.x	Non-food application
Lim et al.	*Opuntia* stricta	2012	-	Review
Ghazi et al.	Fatty acids sterols and vitamin e composition of seed oil of *Opuntia* ficus indica and *Opuntia dillenii* from morocco	2013	-	Chemical characterization
Shahzadi et al.	Synthesis of Some O-Substituted Derivatives of Natural 6-hydroxymethyl-4-methoxy-2H-pyran-2-one (opuntiol)	2013	-	Chemical characterization
Sharkar et al.	Ethnomedicinal importance of the plants in villages in kushtia sador and mirpur upozila, bangladesh	2013	10.1080/10496475.2013.818606	Ethnobotanics
Kanwal et al.	Use of *Opuntia dillenii* Seeds for Sorptive Removal of Acidic Textile Dyes from Water in Benign Way	2013	10.14233/ajchem.2013.14576	Non-food application
Yang et al.	Optimum extraction of polysaccharides from *Opuntia dillenii* and evaluation of its antioxidant activities	2013	10.1016/j.carbpol.2013.05.057	Only antioxidant
Bajaj et al.	Review on research of suppression male fertility and male contraceptive drug development by natural products	2013	10.1177/1934578x1300800840	Review
Anjalam et al.	Exploration of medicinal plants used by the Malayali tribes of Kolli hills, Tamilnadu, India	2014	10.7897/2277-4343.05240	Ethnobotanics

Franchi et al.	Is flower/corolla closure linked to decrease in viability of desiccation-sensitive pollen? Facts and hypotheses: a review of current literature with the support of some new experimental data	2014	10.1007/s00606-013-0911-x	Ethnobotanics
De Jesus	Extraction and Characterization of Essential Oil from *Opuntia* Ficus Indica Seeds	2014	-	Review
Eddouks et al.	Antidiabetic plants improving insulin sensitivity	2014	10.1111/jphp.12243	Review
El Kharrassi et al.	Flowering and fruiting phenology, and physico-chemical characteristics of 2-year-old plants of six species of *Opuntia* from eight regions of Morocco	2015	10.1080/14620316.2015.11668731	Chemical characterization
Jesus Cejudo-Bastante et al.	Potential use of new Colombian sources of betalains. Colorimetric study of red prickly pear (*Opuntia dillenii*) extracts under different technological conditions	2015	10.1016/j.foodres.2015.02.011	Chemical characterization
Jimenez-Aguilar el tal.	Effect of High Hydrostatic Pressure on the Content of Phytochemical Compounds and Antioxidant Activity of Prickly Pears (*Opuntia* ficus-indica) Beverages	2015	10.1007/s12393-015-9111-5	Chemical characterization
Kalegowda et al.	Minerals of cactus (*Opuntia dillenii*): Cladode and fruit	2015	10.18520/v109/i12/2295-2298	Chemical characterization
Mulas et al.	Cladode Biomass Composition of Selected Clones from an *Opuntia* spp. Collection	2015	10.17660/ActaHortic.2015.1067.39	Chemical characterization
Perez Mendez et al.	Physicochemical characterization of cactus pads from *Opuntia dillenii* and *Opuntia* ficus indica	2015	10.1016/j.foodchem.2015.05.011	Chemical characterization
Ragupathi et al.	Selective liquid phase oxidation of benzyl alcohol catalyzed by copper aluminate nanostructures	2015	10.1016/j.molstruc.2014.09.045	Chemical characterization
Xu et al.	Determination of the Volatiles in *Opuntia dillenii* by Headspace Solid-Phase Microextraction and Gas Chromatography-Mass Spectrometry	2015	10.1080/10739149.2015.1004081	Chemical characterization

Ahmad et al.	Ethnopharmacological documentation of medicinal plants used for hypertension among the local communities of DIR Lower, Pakistan	2015	10.1016/j.jep.2015.09.014	Ethnobotanics
Arevalo et al.	Experimental management control of *Opuntia dillenii* Haw. and Agave americana L. in Teno Rural Park, Canary Islands	2015	10.1111/1442-1984.12049	Ethnobotanics
Blasco et al.	Difference in the Invasive Potential of *Opuntia* Genus Inhabiting the South of Iberian Peninsula	2015	0.17660/ActaHortic.2015 .1067.8	Ethnobotanics
Lloret-Salamanca et al.	Comparative Morphological Analysis of Areole and Glochids of Two *Opuntia* Species	2015	10.17660/ActaHortic.201 5.1067.7	Ethnobotanics
Nogales et al.	The unnoticed effect of a top predator on complex mutualistic ecological interactions	2015	10.1007/s10530-014-0823-x	Ethnobotanics
Rashid et al.	Ethnobotanical survey of medicinally important shrubs and trees of Himalayan region of Azad Jammu and Kashmir, Pakistan	2015	10.1016/j.jep.2015.03.042	Ethnobotanics
Ratnaweera et al.	Antimicrobial activities of endophytic fungi obtained from the arid zone invasive plant *Opuntia dillenii* and the isolation of equisetin, from endophytic Fusarium sp.	2015	10.1186/s12906-015-0722-4	Ethnobotanics
Tariq et al.	Ethnomedicinal Evaluation of Medicinal Plants Used against Gastrointestinal Complaints	2015	10.1155/2015/892947	Ethnobotanics
Yaseen et al.	Traditional management of diabetes in Pakistan: Ethnobotanical investigation from Traditional Health Practitioners	2015	10.1016/j.jep.2015.07.041	Ethnobotanics
Khan et al.	Ethnomedicinal uses of Edible Wild Fruits (EWFs) in Swat Valley, Northern Pakistan	2015	10.1016/j.jep.2015.07.029	No *Opuntia dillenii*
Chen et al.	Emulsification and antioxidation of biosurfactant extracts from Chinese medicinal herbs fermentation in vitro	2015	10.1016/j.jbiosc.2015.02. 010	Non-food application
Meng et al.	Effects of massage treatment combined with topical cactus and aloe on puerperal milk stasis	2015	10.3233/BD-150401	Non-food application

Ghazi et al.	Chemical composition and antioxidant activity of seeds oils and fruit juice of *Opuntia* ficus indica and *Opuntia dillenii* from Morocco	2015	-	Only antioxidant
Adnan et al.	Ethnomedicinal plants used against common digestive problems	2015	10.4314/ajtcam.v12i5.15	Review
Shirani et al.	Phytotrapy of cyclophosphamide-induced immunosuppression	2015	10.1016/j.etap.2015.04.012	Review
Han et al.	Extraction optimization by response surface methodology of mucilage polysaccharide from the peel of *Opuntia dillenii* haw. fruits and their physicochemical properties	2016	10.1016/j.carbpol.2016.05.085	Chemical characterization
Montes-Lora et al.	Effect of technological practices on individual betalains and antioxidant activity of Columbian betalain-rich raw materials	2016	10.1111/ijfs.13056	Chemical characterization
Moortheswaran et al.	One-Pot Combustion Synthesis and Characterization Studies of Spinel CoAl2O4 Nano-Catalysts	2016	10.1166/nnl.2016.2147	Chemical characterization
Moortheswaran et al.	Selective Catalytic Oxidation of Benzyl Alcohol and Characterization Studies of Spinel MnAl2O4 Nanoparticles by a Facile Synthesis Route	2016	10.1166/nnl.2016.2146	Chemical characterization
Moussa-Ayoub et al.	Technological characteristics and selected bioactive compounds of *Opuntia dillenii* cactus fruit juice following the impact of pulsed electric field pre-treatment	2016	10.1016/j.foodchem.2016.04.115	Chemical characterization
Ramamoorthy et al.	Betalain and anthocyanin dye-sensitized solar cells	2016	10.1007/s10800-016-0974-9	Chemical characterization
Roome et al.	Opuntiol/Opuntioside-I: a novel suppressor of cytokine, chemokine and lipid mediators in in-vivo and in-vitro inflammatory models	2016	-	Congress communication
Asensi et al.	Alien plants of coastal dune habitats in southern Spain	2016	10.1080/11263504.2014.973463	Ethnobotanics

Castro et al.	CMA band variability and physical mapping of 5S and 45S rDNA sites in Brazilian Cactaceae: Pereskioideae and Opuntioideae	2016	10.1007/s40415-015-0248-5	Ethnobotanics
Gaballah et al.	Changes in physical and chemical properties of *Opuntia dillenii* fruits during the growing stages	2016	10.56890/jpacd.v18i.47	Ethnobotanics
Garrison et al.	Intraspecific variation in opportunistic use of trophic resources by the lizard Ameiva corax (Squamata: Teiidae)	2016	10.1163/15685381-00003060	Ethnobotanics
Holmes	Morphological and ecological characterization of *Opuntia* Miller on the coast of South Carolina	2016	-	Ethnobotanics
Pignotti et al.	Effect of nopales (*Opuntia* spp.) on lipoprotein profile and oxidative stress among moderately hypercholesterolemic adults: A pilot study	2016	10.1016/j.jff.2016.08.060	No *Opuntia dillenii*
Li et al.	Extraction of *Opuntia dillenii* Haw. Polysaccharides and Their Antioxidant Activities	2016	10.3390/molecules21121612	Only antioxidant
Ciriminna et al.	*Opuntia* ficus-indica seed oil: Biorefinery and bioeconomy aspects	2017	10.1002/ejlt.201700013	Chemical characterization
Kalegowda et al.	*Opuntia dillenii* (Ker-gawl) haw fruit peel pectin: Physicochemical, rheological, and functional behavior	2017	10.1111/jfpp.13165	Chemical characterization
Kalegowda et al.	*Opuntia dillenii* (Ker-Gawl) Haw cladode mucilage: Physico-chemical, rheological and functional behavior	2017	10.1016/j.carbpol.2016.10.070	Chemical characterization
Moussa-Ayoub et al.	Impact of pulsed electric fields, high hydrostatic pressure, and thermal pasteurization on selected characteristics of *Opuntia dillenii* cactus juice	2017	10.1016/j.lwt.2016.10.061	Chemical characterization
Reis et al.	Bioactive compounds and morphology in *Opuntia* spp. fruits from Portuguese ecotypes	2017	-	Ethnobotanics
Betancourt et al.	Pigment composition and antioxidant capacity of betacyanins and betaxanthins fractions of *Opuntia dillenii* (Ker Gawl) Haw cactus fruit	2017	10.1016/j.foodres.2017.09.007	Only antioxidant

Li et al.	Food-derived antioxidant polysaccharides and their pharmacological potential in neurodegenerative diseases	2017	10.3390/nu9070778	Review
Raimundo et al.	Rapid, simple and potentially universal method for DNA extraction from *Opuntia* spp. fresh cladode tissues suitable for PCR amplification	2018	10.1007/s11033-018-4303-8	Chemical characterization
Rayan et al.	Enrichment of rice-based extrudates with Cactus *Opuntia dillenii* seed powder: a novel source of fiber and antioxidants	2018	10.1007/s13197-017-2961-5	Combined use
Garcia-de-Lomas et al.	Mechanical and manual control of prickly pear *Opuntia dillenii* in lakeside dunes at Laguna del Portil, Southern Spain	2018	-	Ethnobotanics
Reis et al.	Assessment of Genetic Diversity in *Opuntia* spp. Portuguese Populations Using SSR Molecular Markers	2018	10.3390/agronomy8040055	Ethnobotanics
Reis et al.	Characterization and Evaluation of Portuguese *Opuntia* spp. Germplasm	2018	10.13140/RG.2.2.34992.87040	Ethnobotanics
Tamba et al.	Coupling of pressure-driven membrane technologies for concentrating, purifying and fractionizing betacyanins in cactus pear (*Opuntia dillenii* Haw.) juice	2019	10.1016/j.ifset.2018.12.008	Chemical characterization
Thu Minh et al.	Profiling of polar metabolites in fruits of *Opuntia* stricta var. dillenii by ion-pair high-performance countercurrent chromatography and off-line electrospray mass-spectrometry injection	2019	10.1016/j.chroma.2019.06.009	Chemical characterization
De la Riva et al.	Functional and phylogenetic consequences of plant invasion for coastal native communities	2019	10.1111/jvs.12748	Ethnobotanics
Fukatsu et al.	Hybridization, coexistence, and possible reproductive interference between native Oxalis corniculata and alien O-dillenii in Japan	2019	10.1007/s00606-018-1557-5	Ethnobotanics

Chauhan et al.	Development of spiced squash (appetizer) from wild prickly pear (*Opuntia dillenii* Haw.) and its quality evaluation during storage	2019	10.31018/jans.v11i2.2049	Food technology
Wu	Effect of *Opuntia dillenii* polysaccharide on gelling properties of Trichiurus lepturus myobrilar protein	2019	10.1016/j.ijbiomac.2019.03.024	Food technology
Wu	Extending shelf-life of fresh-cut potato with cactus *Opuntia dillenii* polysaccharide-based edible coatings	2019	10.1016/j.ijbiomac.2019.03.022	Food technology
Ali et al.	Extraction and Identification of Cactus *Opuntia dillenii* Seed Oil and its Added Value for Human Health Benefits	2019	10.5530/pj.2019.11.93	Only antioxidant
Habtemariam et al.	The chemical and pharmacological basis of prickly pear cactus (*Opuntia* species) as potential therapy for type 2 diabetes and obesity	2019	10.1016/B978-0-08-102922-0.00013-4	Review
Shirazinia et al.	*Opuntia dillenii*: A Forgotten Plant with Promising Pharmacological Properties.	2019	10.3831/KPI.2019.22.002	Review
El Mokni el al.	New records of cacti (Opuntioideae & Cactoideae, Cactaceae) from Tunisia	2020	10.25223/brad.n38.2020.a6	Ethnobotanics
Fraga-Arguimbau et al.	Notes and Contributions to the Flora Of Menorca (Xv). A Contribution to the Alien Flora	2020	-	Ethnobotanics
Parveen et al.	Accumulation of selected metals in the fruits of medicinal plants grown in urban environment of Islamabad, Pakistan	2020	10.1016/j.arabjc.2017.04.010	Ethnobotanics
Kumar et al.	Aloe trinervis sp. nov.: A new succulent species from Indian Desert (Asphodelaceae)	2020	10.1016/j.japb.2020.03.001	No *Opuntia dillenii*
Bassama et al.	Degradation Kinetics of Betacyanins during the Pasteurization and Storage of Cactus Pear (*Opuntia dillenii* Haw.) Juice Using the Arrhenius, Eyring, and Ball Models	2021	10.3390/beverages7010002	Chemical characterization
Castellano et al.	*Opuntia* spp. Fibre Characterisation to Obtain Sustainable Materials in the Composites Field	2021	10.3390/polym13132085	Chemical characterization

Gomez-Lopez et al.	Characterization, Stability, and Bioaccessibility of Betalain and Phenolic Compounds from *Opuntia* stricta var. Dillenii Fruits and Products of Their Industrialization	2021	10.3390/foods10071593	Chemical characterization
Lakshme et al.	Preliminary Phytochemical Screening and Estimation of Total Phenolic Content of Aqueous Cladode Extract of *Opuntia dillenii*	2021	-	Chemical characterization
Loukili et al.	Chemical Composition and Physicochemical Analysis of *Opuntia dillenii* Extracts Grown in Morocco	2021	10.1155/2021/8858929	Chemical characterization
Nguyen et al.	Extraction and Physico-Chemical Characterization of Water-Soluble Polysaccharides From *Opuntia dillenii* (Ker Gawl.) Haw. Cladodes	2021	10.15625/2525-2518/59/2/15510	Chemical characterization
Siddiqui et al.	Toxicological assessment of *Opuntia dillenii* (Ker Gawl.) Haw. cladode methanol extract, fractions and its alpha pyrones: Opuntiol and opuntioside	2021	10.1016/j.jep.2021.114409	Chemical characterization
Surup et al.	Opuntisines, 14-membered cyclopeptide alkaloids from fruits of *Opuntia* stricta var. dillenii isolated by high-performance countercurrent chromatography	2021	10.1016/j.foodchem.2020.127552	Chemical characterization
Galappaththi et al.	Cochineal Scale Dactylopius *Opuntia*e controls *Opuntia dillenii* in Bundala National Park, Sri Lanka	2021	10.4038/cjs.v50i3.7912	Ethnobotanics
Groom et al.	Hybridization of Oxalis corniculata and *O. dillenii* in their non-native range	2021	10.3897/phytokeys.178.61031	Ethnobotanics
Majeed et al.	Ethnobotany, medicinal utilization and systematics of *Opuntia* species from deserts of Pakistan	2021	10.1007/978-3-030-78444-7_3	Ethnobotanics
Prabhu et al.	An ethnobotanical study of medicinal plants used in pachamalai hills of Tamil Nadu, India	2021	10.1016/j.hermed.2020.100400	Ethnobotanics
Saldana-Lopez et al.	Assembly of species' climatic niches of coastal communities does not shift after invasion	2021	10.1111/jvs.12989	Ethnobotanics
Ye et al.	Medicinal Angiosperms of Begoniaceae, Cactaceae, and Theaceae	2021	10.1007/978-981-16-5880-8_3	Ethnobotanics

Madushan et al.	Use of natural plant extracts as a novel microbiological quality indicator in raw milk: An alternative for resazurin dye reduction method	2021	10.1016/j.lwt.2021.111221	Food technology
Bezerril et al.	Physicochemical characteristics and bioactive compounds of the Xique-xique (Pilosocereus gounellei) cactus from Caatinga Brazilian: are they nutritive and functional?	2021	10.1007/s11694-021-00906-w	No *Opuntia dillenii*
Penuela et al.	Behavior, ecology and territory of the chestnut-bellied hummingbird, Saucerottia castaneiventris, in the xerophytic vegetation of the Chicamocha canyon of Colombia	2021	10.3897/neotropical.16.e66094	No *Opuntia dillenii*
Rehioui et al.	Corrosion inhibiting effect of a green formulation based on *Opuntia dillenii* seed oil for iron in acid rain solution	2021	10.1016/j.heliyon.2021.e06674	Non-food application
Alsaad et al.	Study of the antioxidant activity of cactus (*Opuntia* dellienii) fruits (pulp and peels) and characterisation of their bioactive compounds by gc-ms	2021	10.37077/25200860.2021.34.2.16	Only antioxidant
Gomez-Marqueo et al.	In Vitro Antioxidant Capacity of *Opuntia* spp. Fruits Measured by the LOX-FL Method and its High Sensitivity Towards Betalains	2021	10.1007/s11130-021-00914-7	Only antioxidant
Bouhrim et al.	Phytochemistry and biological activities of *Opuntia* seed oils: *Opuntia dillenii* (Ker Gawl.) Haw. And *Opuntia* ficus-indica (L.) Mill. A review	2021	10.2478/hepo-2021-0008	Review
Daoudi et al.	A review on hepatoprotective effects of some medicinal plant oils	2021	10.2174/1570180817666200831175139	Review
Das et al.	Cactus: Chemical, nutraceutical composition and potential bio-pharmacological properties	2021	10.1002/ptr.6889	Review
Raimi et al.	Bioprospecting of endophytic microorganisms for bioactive compounds of therapeutic importance	2021	10.1007/s00203-021-02256-z	Review
Sharifi-Rad et al.	Therapeutic Potential of Neoechinulins and Their Derivatives: An Overview of the Molecular Mechanisms Behind Pharmacological Activities	2021	10.3389/fnut.2021.664197	Review

Bassama et al.	Modeling betacyanin degradation to optimize the quality of pasteurized cactus pear (*Opuntia dillenii* Haw.) juice	2022	10.17660/ActaHortic.2022.1348.3	Chemical characterization
Vazquez-Espinosa et al.	Ultrasound-Assisted Extraction of Betalains from *Opuntia* Fruit Pulp of Different Color Varieties	2022	10.3390/agronomy12112604	Chemical characterization
Yadiki et al.	Temperature and Exposure Time Impact on the Extraction of *Opuntia* ficus-indica and *Opuntia dillenii* Cladodes on % Yield as a Response: Screening using Expert Software	2022	10.25259/GJMPBU_55_2022	Chemical characterization
Nizamuddin et al.	Plant chemical analysis and antioxidant commotion of lactobacillus contrived hydroethanolic extracts of *Opuntia dillenii* Haw.	2022	10.54085/ap.2022.11.2.61	Combined use
Besne-Eseverri et al.	Effects of *Opuntia* stricta var. dillenii extracts on in vitro triglyceride accumulation prevention in mouse AML-12 hepatocytes	2022	-	Congress communication
Gomez-Lopez et al.	Characterization, bioaccessibility and biological activities of betalains and phenolic compounds from *Opuntia* stricta var. Dillenii fruits	2022	-	Congress communication
Akroud et al.	Genetic relations among Moroccan *Opuntia* genotypes with different degrees of resistance to Dactylopius *Opuntia*e	2022	10.56890/jpacd.v24i.480	Ethnobotanics
Almeida et al.	Selection of *Opuntia* spp. accessions with resistance to false cochineal carmine (Dactylopius *Opuntia*e)	2022	10.17660/ActaHortic.2022.1343.2	Ethnobotanics
Guiggi et al.	Taxonomic and nomenclatural novelties in some Cactaceae of Greater Antilles	2022	10.11646/phytotaxa.573.2.3	Ethnobotanics
Majeed et al.	Morpho-palynological and anatomical studies in desert cacti (*Opuntia dillenii* and *Opuntia* monacantha) using light and scanning electron microscopy	2022	10.1002/jemt.24129	Ethnobotanics
Rehman et al.	Ethnogynaecological Knowledge of Traditional Medicinal Plants Used by the Indigenous Communities of North Waziristan, Pakistan	2022	10.1155/2022/6528264	Ethnobotanics

Sen et al.	Climate resilient wild edible succulents of Mandi Himachal Pradesh, their indigenous uses and role in socio-economic development of the region	2022	10.1007/978-981-19-5061-2_18	Ethnobotanics
Zaman et al.	A Preliminary Checklist, Phenology, and Biological Spectrum of the Vascular Flora of Manglot Wildlife Park, Nizampur, Pakistan	2022	10.1007/978-3-030-73943-0_23	Ethnobotanics
Ayesha et al.	Supplementation of unleavened flat-bread with lotus root powder exhibits promising antioxidant, anti-inflammatory and analgesic effects: A study involving biochemical and in vivo approach	2022	10.36721/PJPS.2022.35.6.REG.1615-1625.1	No *Opuntia dillenii*
Choi et al.	First report of powdery mildew caused by Erysiphe russellii on Oxalis dillenii in Korea	2022	10.1007/s42161-022-01062-w	Non-food application
Mohamed Nijaz et al.	Antilarval and in vitro Anticancer efficacy of Cladode extracts of *Opuntia dillenii* (Ker Gawl.) Haw., Cereus pterogonus Lem. and Acanthocereus tetragonus (L.) Hummelinck	2022	10.52711/0974-360X.2022.00480	Non-food application
Gomez-Lopez et al.	Pressurized green liquid extraction of betalains and phenolic compounds from *Opuntia* stricta var. Dillenii whole fruit: Process optimization and biological activities of green extracts	2022	10.1016/j.ifset.2022.103066	Only antioxidant
Vieira et al.	Correlation and influence of antioxidant compounds of peels and pulps of different species of cacti from Brazilian Caatinga biome using principal component analysis	2022	10.1016/j.sajb.2022.02.005	Only antioxidant
Alqaktani et al.	Bioactive Constituents and Toxicological Evaluation of Selected Antidiabetic Medicinal Plants of Saudi Arabia	2022	10.1155/2022/7123521	Review
Barbhuiya et al.	Ethnomedicinal Practices and Traditional Medicinal Plants of Barak Valley, Assam: a systematic review	2022	10.3831/KPI.2022.25.3.149	Review
Bastola et al.	*Opuntia dillenii* (Ker Gawl.) Haw	2022	10.1016/B978-0-323-85591-4.00009-X	Review

Jiang et al.	Bioactive polysaccharides and their potential health benefits in reducing the risks of atherosclerosis: A review	2022	10.1111/jfbc.14337	Review
Yang et al.	Research progress on extraction, purification, analysis and biological activity of polysaccharides from *Opuntia dillenii* Haw	2022	10.13652/j.spjx.1003.5788.2022.90114	Review
Al-Naqeb et al.	Supercritical Fluid Extraction of Oils from Cactus *Opuntia* ficus-indica L. and *Opuntia dillenii* Seeds	2023	10.3390/foods12030618	Chemical characterization
Morales-De la Pena et al.	Application of moderate intensity pulsed electric fields in red prickly pears and soymilk to develop a plant-based beverage with potential health-related benefits	2023	10.1016/j.ifset.2023.103421	Chemical characterization
Parralejo-Sanz et al.	Oil-Based Double Emulsion Microcarriers for Enhanced Stability and Bioaccessibility of Betalains and Phenolic Compounds from *Opuntia* stricta var. dillenii Green Extracts	2023	10.3390/foods12112243	Chemical characterization
Taheur et al.	Functional properties of a kefir-based probiotic dairy product enriched with red prickly pear (*Opuntia dillenii*) powder	2023	10.1007/s11694-023-02136-8	Combined use
Alharthi et al.	Biological change of western Saudi Arabia: Alien plants diversity and their relationship with edaphic variables	2023	10.1016/j.jksus.2022.102496	Ethnobotanics
Guerrero-Campos et al.	Introduced rabbits as seed-dispersing frugivores: a study case on a environmentally diverse oceanic island (Tenerife, Canaries)	2023	10.1007/s10530-023-03026-2	Ethnobotanics
Hoshino et al.	Long-styled Variant Decreases Ovule Loss by Hybridization with Short-styled Congener in Oxalis	2023	10.18942/apg.202309	Ethnobotanics
Wu et al.	Effects of cactus *Opuntia dillenii* polysaccharide-based coatings loaded with glutathione on the preservation of freshly cut Chinese water chestnut	2023	10.1016/j.foodchem.2022.134187	Food technology
Chiu et al.	Mechanism and inhibitory effects of cactus (*Opuntia dillenii*) extract on melanocytes and its potential application for whitening cosmetics	2023	10.1038/s41598-022-26125-x	Non-food application

Cotino et al.	Antioxidant activity of two *Opuntia* Mill. species fruit extracts on human sperm quality after a freeze-thaw cycle	2023	10.1080/14786419.2022.2124984	Non-food application
El Aalaoui et al.	Potential of parasitoids to control Diaspis echinocacti (Bouche) (Hemiptera: Diaspididae) on *Opuntia* spp. cactus pear	2023	10.1186/s41938-023-00704-0	Non-food application
Prasad et al.	Role of sugarcane bagasse biogenic silica on cellulosic *Opuntia dillenii* fibre-reinforced epoxy resin biocomposite: mechanical, thermal and laminar shear strength properties	2023	10.1007/s13399-021-02154-w	Non-food application
Rehioui et al.	Development of a promising nontoxic corrosion inhibitor based on *Opuntia dillenii* seed oil for iron corrosion in 3Wt% NaCl: Experimental and theoretical approaches	2023	10.1016/j.cdc.2023.101037	Non-food application
Besne-Eseverri et al.	Beneficial Effects of *Opuntia* spp. on Liver Health	2023	10.3390/antiox12061174	Review
Gandhi et al.	Anti-inflammatory natural products modulate interleukins and their related signaling markers in inflammatory bowel disease: A systematic review	2023	10.1016/j.jpha.2023.09.012	Review
Kumari et al.	Antimicrobial Activity of Bioactive Compounds Isolated from Plant Endophytes	2023	10.2174/1573407218666220524120648	Review
Lu et al.	Recent Research on Different Parts and Extracts of *Opuntia dillenii* and Its Bioactive Components, Functional Properties, and Applications	2023	10.3390/nu15132962	Review
Saklani et al.	Ethnomedicinal importance of some bee floral resources of Shivalik hills of Himachal Pradesh: a review	2023	10.5958/0974-4576.2023.00079.8	Review
Cruz et al.	Semisynthesis of Betaxanthins from Purified Betacyanin of *Opuntia dillenii* sp.: Color Stability and Antiradical Capacity	2024	10.3390/molecules29092116	Chemical characterization
Dhanapal et al.	Standardization and HPTLC Fingerprint Studies on the Ethanol Extracts of Vitex negundo Leaves and *Opuntia dillenii* Cladode	2024	10.18311/jnr/2024/42111	Chemical characterization

Di Napoli et al.	Chemical composition, antimicrobial, and antioxidant activities of *Opuntia* stricta (Haw.) Haw. mucilage collected in Sicily, Italy	2024	10.1080/14786419.2023.2272781	Chemical characterization
Elouazkiti el al.	Proximate nutritive values, antioxidant capacity, antimicrobial properties and profiling the chemical content of *Opuntia* stricta cladodes and fruit peel by HPLC-DAD-ESI/MS analyses	2024	10.1080/14786419.2024.2381028	Chemical characterization
Kallel et al.	By-product of prickly pear juice industry: a novel ingredient to improve the physicochemical, texture and sensory properties of cake	2024	10.1007/s11694-024-02756-8	Chemical characterization
Liu et al.	Comparative study on chain conformations, physicochemical and rheological properties of three acidic polysaccharides from *Opuntia dillenii* Haw. fruits	2024	10.1016/j.ijbiomac.2024.130027	Chemical characterization
Martin et al.	Unlocking the potential of *Opuntia* species mucilage in chemistry	2024	10.1016/j.ijbiomac.2024.131647	Chemical characterization
Zazouli et al.	Morphological and biochemical characterization of Moroccan *Opuntia dillenii* fruit: Natural source of bioactive compounds	2024	10.17533/udea.vitae.v31n2a353392	Chemical characterization
Alvarado-Lopez et al.	A Healthy Brazil Nut Beverage with *Opuntia* stricta var. dillenii Green Extract: Beverage Stability and Changes in Bioactives and Antioxidant Activity during Cold Storage	2024	10.3390/foods13081237	Combined use
El Hassania el al.	Physicochemical and phytochemical characterization of *Opuntia dillenii*: A promising source of bioactive compounds	2024	10.1080/10942912.2024.2385960	Ethnobotanics
Marhri et al.	A Comparative Analysis of Morphological Characteristics between Endangered Local Prickly Pear and the Newly Introduced Dactylopius *Opuntia*e-Resistant Species in Eastern Morocco	2024	10.1155/2024/7939465	Ethnobotanics
Munne-Bosch et al.	The dramatic effects of well-intentioned but ill-designed management strategies in plant biological invasions	2024	10.1038/s41477-024-01747-3	Ethnobotanics

Singhal et al.	Ethnoveterinary plants of Renuka tehsil in Sirmour district of Himachal Pradesh (India)	2024	10.32859/era.29.3.1-22	Ethnobotanics
Suraweera et al.	Silvicultural Practices for Diversity Conservation and Invasive Species Suppression in Forest Ecosystems of the Bundala National Park, Sri Lanka	2024	10.3390/plants13010121	Ethnobotanics
Yin et al.	Lipid metabolism regulation by dietary polysaccharides with different structural properties	2024	10.1016/j.ijbiomac.2024. 132253	No *Opuntia dillenii*
Liu et al.	Structural characterization of three acidic polysaccharides from *Opuntia dillenii* Haw. fruits and their protective effect against hydrogen peroxide-induced oxidative stress in Huh-7 cells	2024	10.26599/FSHW.2022.92 50160	Only antioxidant
Marhri et al.	Comparative Analysis of Antioxidant Potency and Phenolic Compounds in Fruit Peel of *Opuntia* robusta, *Opuntia dillenii*, and *Opuntia* ficus-indica Using HPLC-DAD Profiling	2024	10.1155/2024/2742606	Only antioxidant
Marhri et al.	HPLC-DAD Profiling of Phenolic Components and Comparative Assessment of Antioxidant Potency in *Opuntia* robusta, *Opuntia dillenii*, and *Opuntia* ficusindica Cladodes at Diverse Stages of Ripening	2024	10.5650/jos.ess24034	Only antioxidant
Rehioui et al.	Physicochemical Properties of Moroccan *Opuntia dillenii* Fruit, Extraction of Betacyanins and Study of its Stability and Antioxidant Activity	2024	10.55251/jmbfs.9098	Only antioxidant
Rhizlan et al.	In vitro Study on Antioxidant and Antiglycation Activities, and Molecular Docking of Moroccan Medicinal Plants for Diabetes	2024	10.2174/22150838106662 30831154738	Only antioxidant
Rocha et al.	Exploring the potential prebiotic effects of *Opuntia dillenii* (Ker Gawl). Haw (Cactaceae) cladodes on human intestinal microbiota	2024	10.1016/j.jff.2024.106259	Only antioxidant

Hudu et al.	Dissecting the low morbidity and mortality during the COVID-19 pandemic in Africa: a critical review of the facts and fallacies	2024	10.1007/s13596-023-00739-6	Review
Loukili et al.	Phytochemical, biological, and nutritional properties of the prickly pear, *Opuntia dillenii*: : A review	2024	10.1016/j.jsps.2024.102167	Review
De Araujo et al.	Impact of different prebiotics on ultrasound-treated nopal cladode (*Opuntia dillenii*) beverages	2025	10.1016/j.foodchem.2024.141721	Combined use
Mehta et al.	Postharvest integration of prickly pear betalain-enriched gummies with different sugar substitutes for decoding diabetes type-II and skin resilience- in vitro and in silico study	2025	10.1016/j.foodchem.2024.141612	Combined use
Ennouri et al.	Drinkable beverage based on whey and *Opuntia* stricta (var. dillenii) juice derived from underexploited biowaste: from process development to characterization	2025	10.1007/s13399-023-04188-8	Food technology
Muzammal et al.	Eco-friendly Cr2O3 nanoparticles from *Opuntia dillenii* for visible-light photocatalysis and antimicrobial defense against waterborne pathogens	2025	10.1016/j.ceramint.2024.11.490	Non-food application
Parralejo-Sanz et al.	Assessment of the immunomodulatory potential of betalain- and phenolic-rich extracts from *Opuntia* cactus fruits	2025	10.1016/j.fbio.2025.106093	Only antioxidant
Cui et al.	Preparing the compound of chiral 2-hydroxyl acenaphthenone comprises putting crushed fresh plant and water into a reactor, adding an acenaphthenone compound to the reactor, adding diatomite to the reaction system, and stirring and filtering	-	-	Patent
Guo et al.	Traditional Chinese medicine of compounded chlorophyll for treating vegetative nervous disorder	-	-	Patent
Ki et al.	Method of making nutrient-enriched fermented soy products using medicinal herbs and fruits together with fermented soybeans	-	-	Patent

Kim et al.	Production of mixed grain powder which is rich in vitamin c and dietary fiber and contains efficacy of cactus by freeze drying and grinding cactus and then mixing with cereal powder	-	-	Patent
Jung et al.	Laver has multiple patterns, which are prepared by sprinkling natural powder on rectangular wet laver to form pattern like character, figure and logo and patterns are dried	-	-	Patent
Kim et al.	Chemical-free and antibiotic bean-sprouts using herbal medicine and method for cultivation	-	-	Patent
Ceng et al.	Preparation of wild *Opuntia dillenii* polysaccharide extract e.g. for treating fatty liver, by removing wild *Opuntia dillenii* thorns and peeling, crushing, leaching, centrifuging, vacuum-condensing, centrifuging, condensing and freeze-drying	-	-	Patent
Choi et al.	Method of making functional dried fish product or salted fish using glasswort extract, propolis, citrus junos tanaka extract and *Opuntia dillenii* extract	-	-	Patent
Kim et al.	Production of red pepper paste using *Opuntia dillenii* haw. as main component and red pepper paste having excellent taste and tackiness prepared thereby	-	-	Patent
Kwon et al.	Production of *Opuntia dillenii* red pepper paste eliminated in hot taste while maintaining sourish taste of *Opuntia dillenii* and tackiness and viscosity of red pepper paste by adding *Opuntia dillenii* powder to conventional red pep	-	-	Patent
Lee et al.	Production of lactic acid fermented beverage by inoculation of lactic acid bacteria starter into culture liquid containing *Opuntia dillenii*, liquid fructose and yeast extract and fermenting	-	-	Patent

Mi et al.	Preparing functional pork cutlet, by kneading vegetable juice from vegetables with wheat flour to obtain dough, placing dough on mold pate to form character and logo member of desired shapes, freezing and attaching with sliced pork	-	-	Patent
Oh et al.	Rose tea composition containing vegetable roses, *Opuntia dillenii*, lycium chinense and jasmine having excellent taste and flavor	-	-	Patent
Pack et al.	Method for manufacturing Ulmus davidiana juice useful for treating e.g. cancer and diabetes	-	-	Patent
Park et al.	Method of making long white rice cake with various colors using rice, raspberry, persimmon, mugwort, *Opuntia* and sweet pumpkin	-	-	Patent
Park et al.	Citrus peel tea containing carrot juice prepared by mixing carrot juice, citrus sunki peel powder and white sugar	-	-	Patent
Park et al.	Rice wrapped in seaweed having various colors using paprika, mugwort, pine pollen and *Opuntia dillenii*	-	-	Patent
Shin et al.	Method of making fermented milk for pig by using fruit powder of *Opuntia dillenii* and lactic acid bacteria	-	-	Patent
Sung et al.	Method of making glutinous rice cake using fruit powder of *Opuntia dillenii* and glutinous rice flour	-	-	Patent
Yang et al.	Natural honey tea using extract of sasa quelpaertensis nakai and citrus unshiu and preparation method thereof	-	-	Patent
Youn et al.	Preparation of health beverage having excellent efficacy on asthma, cold, cough, phlegm by using sponge gourd, pear and balloon flower as main material	-	-	Patent
Zhu et al.	Extraction of sticky biological stock solution and its uses	-	-	Patent

Choi et al.	Method for manufacturing tonic noodles and noodle products manufactured thereby	-	-	Patent
Kim et al.	Preparation of health food in form of tablet using ostrich eggs as main component	-	-	Patent

Table S2. Risk of bias assessment in animal studies based on the SYRCLE's tool

Reference	Type of bias									
	Selection bias			Performance bias		Detection bias		Attrition bias	Reporting bias	Other sources of bias
	Sequence generation	Baseline characteristics	Allocation concealment	Random housing	Blinding	Random outcome assessment	Blinding	Incomplete outcome data	Selective outcome reporting	
[15]	Yes	Yes	Unclear	Yes	Unclear	Unclear	Unclear	No	No	Yes
[16]	Yes	Yes	Unclear	Yes	Unclear	Unclear	Unclear	No	No	Yes
[17]	Yes	Yes	Unclear	Yes	Unclear	Unclear	Unclear	No	No	Yes
[18]	Unclear	Unclear	Unclear	Unclear	Unclear	Unclear	Unclear	Yes	No	Yes
[19]	Yes	Yes	Unclear	Unclear	Unclear	Unclear	Unclear	No	No	No
[22]	No	Yes	No	No	No	Unclear	Unclear	No	No	Yes
[28]	Yes	Yes	Yes	Yes	Yes	Unclear	Unclear	No	No	Yes
[29]	Yes	Yes	Yes	Yes	Unclear	Yes	Unclear	No	No	No
[30]	Yes	Yes	Yes	Unclear	Unclear	Unclear	Unclear	No	No	No
[31]	Yes	Yes	Unclear	Unclear	Unclear	Unclear	Unclear	No	No	Yes
[32]]	Yes	Yes	Unclear	Unclear	Unclear	Unclaer	Unclear	No	No	Yes
[33]	Yes	Yes	Unclear	Unclear	Unclear	Unclear	Unclear	No	No	Yes
[34]	Yes	Yes	Unclear	Unclear	Unclear	Unclear	Unclear	No	No	Yes
[35]	Yes	Yes	Unclear	Unclear	Unclear	Unclear	Unclear	No	No	Yes
[36]	Yes	Yes	Unclear	Unclear	Unclear	Unclear	Unclear	No	No	No
[39]	Yes	Yes	Unclear	Unclear	Unclear	Unclear	Unclear	No	No	Yes
[40]	Yes	Yes	Unclear	Unclear	Unclear	Unclear	Unclear	No	No	Yes
[41]	Yes	Yes	Unclear	Yes	Unclear	Unclear	Unclear	No	No	Yes
[42]	Yes	Yes	Unclear	Yes	Unclear	Unclear	Unclear	No	No	Yes
[43]	Yes	Yes	Unclear	Yes	Unclear	Unclear	Unclear	No	No	Yes
[44]	Yes	Yes	Unclear	Yes	Unclear	Unclear	Unclear	No	No	Yes

Table S3. Risk of bias assessment in cell culture studies based on the SYRCLE's tool

Reference	Type of bias									
	Selection bias			Performance bias		Detection bias		Attrition bias	Reporting bias	Other sources of bias
	Sequence generation	Baseline characteristics	Allocation concealment	Random housing	Blinding	Random outcome assessment	Blinding	Incomplete outcome data	Selective outcome reporting	
[12]	Unclear	Yes	N/A	N/A	Unclear	N/A	Unclear	Unclear	Yes	Yes
[20]	Unclear	Yes	N/A	N/A	Unclear	N/A	Unclear	Unclear	Yes	Yes
[21]	Unclear	Yes	N/A	N/A	Unclear	N/A	Unclear	Unclear	Yes	Yes
[22]	Unclear	Yes	N/A	N/A	Unclear	N/A	Unclear	Unclear	Yes	Yes
[23]	Unclear	Yes	N/A	N/A	Unclear	N/A	Unclear	Unclear	Yes	Yes
[24]	Unclear	Yes	N/A	N/A	Unclear	N/A	Unclear	Unclear	Yes	Yes
[25]	Unclear	Yes	N/A	N/A	Unclear	N/A	Unclear	Unclear	Yes	Yes
[26]	Unclear	Yes	N/A	N/A	Unclear	N/A	Unclear	Unclear	Yes	Yes
[27]	Unclear	Yes	N/A	N/A	Unclear	N/A	Unclear	Unclear	Yes	Yes
